# A murine model of Barth syndrome recapitulates human cardiac and skeletal muscle phenotypes

**DOI:** 10.1242/dmm.052077

**Published:** 2025-05-19

**Authors:** Erika Yazawa, Erin M. Keating, Suya Wang, Mason E. Sweat, Qing Ma, Yang Xu, Michael Schlame, William T. Pu

**Affiliations:** ^1^ Division of Newborn Medicine, Department of Pediatrics, Boston Children's Hospital, Boston, MA 02115, USA; ^2^ Department of Cardiology, Boston Children's Hospital, Boston, MA 02115, USA; ^3^ Department of Anesthesiology, New York University School of Medicine, New York, NY 10016, USA

**Keywords:** Tafazzin, Cardiomyopathy, Skeletal myopathy, Barth syndrome

## Abstract

Barth syndrome is a mitochondrial disorder with hallmarks of cardiac and skeletal muscle weakness. It is caused by pathogenic variants in the X-linked gene tafazzin (*TAZ*), required for cardiolipin remodeling. Previously described germline and conditional *Taz* knockout models are not ideal for therapeutic development because they lack the combination of robust survival to adulthood, cardiomyopathy and skeletal muscle weakness. We characterized a cardiac and skeletal muscle-specific *Taz* knockout model (*Taz^mKO^*) in which Cre recombinase is expressed from the muscle creatine kinase promoter (*mCK-Cre*). *Taz^mKO^* mice survived normally. Cardiolipin composition was abnormal in both heart and skeletal muscle. *Taz^mKO^* had reduced heart function by 2 months of age, and function progressively declined thereafter. Reduced treadmill endurance and diminished peak oxygen consumption were evident by 3 months of age, suggesting reduced skeletal muscle function. Electron microscopy showed abnormalities in mitochondrial structure and distribution. Overall, *Taz^mKO^* mice display diminished cardiac function and exercise capacity while maintaining normal survival. This model will be useful for studying the effects of TAZ deficiency in striated muscles and for testing potential therapies for Barth syndrome.

## Teaser

**Summary:** Loss of function of tafazzin causes Barth syndrome. We characterized a murine model with selective skeletal and cardiac knockout of tafazzin that should prove useful for pre-clinical therapeutic development.

## INTRODUCTION

Barth syndrome is an X-linked mitochondrial disorder characterized by cardiac and skeletal myopathy (Clarke et al., 2013). It is caused by pathogenic variants in the gene tafazzin (*TAZ*) (Bione et al., 1996), which encodes a cardiolipin transacylase that catalyzes the exchange of cardiolipin acyl chains. Cardiolipin plays critical roles in maintaining the structural integrity of the inner mitochondrial membrane (Ikon and Ryan, 2017; Joubert and Puff, 2021) and the activity of the electron transport chain (Dudek et al., 2016; Pfeiffer et al., 2003), regulating apoptosis (Kagan et al., 2005) and mitochondrial dynamics (Wang et al., 2023; Zhang et al., 2022).

Currently, there are no targeted therapies for Barth syndrome (Thompson et al., 2022). Development of potential therapies, such as adeno-associated virus-mediated expression of TAZ (Suzuki-Hatano et al., 2019; Wang et al., 2020), require an appropriate animal model (Pu, 2022). In the pure C57BL/6J background, *Taz* germline knockout (*Taz-KO*) mice are small and have low survival beyond the neonatal period (Wang et al., 2020, 2023). The mice that do survive develop both cardiac and skeletal myopathy (Wang et al., 2020, 2023). This low survival made this model suboptimal for studies of adult mice, including therapeutic trials. When crossed with other inbred strains, the F1 *Taz* knockout progeny had normal survival and variable degrees of cardiomyopathy or skeletal myopathy, pointing to an important role for genetic modifiers in the expression of Barth syndrome phenotypes (Wang et al., 2023). However, in different inbred strains, cardiac and skeletal myopathy were often dissociated. For instance, CAST[F1] *Taz-KO* mice had severe cardiomyopathy but normal treadmill endurance, whereas WSB[F1] *Taz-KO* mice had significantly impaired treadmill endurance and mild, late-onset cardiomyopathy. Other than C57BL/6J, no tested genetic background yielded normal survival and both severe skeletal and cardiac muscle phenotypes.

An alternative approach to obtaining a *Taz* knockout mouse model for therapeutic trials is conditional gene inactivation using the Cre-loxP system. Previously, we showed that, in the C57BL/6J background, inactivation of a floxed *Taz* allele by cardiomyocyte-selective *Myh6-Cre* resulted in severe systolic dysfunction by 3 months of age, while survival was normal (Wang et al., 2020). However, this model lacked *Taz* deficiency in skeletal muscles.

To obtain a more suitable mouse model for developing new therapeutics for Barth syndrome, we here characterized *mCK-Cre* conditional *Taz* knockout mice, in which the cardiac and skeletal muscle selective *mCK-Cre* transgene (Brüning et al., 1998) catalyzes inactivation of the floxed *Taz* allele (Ren et al., 2019; Wang et al., 2020).

## RESULTS

### *Taz^mKO^* survival, growth and body composition

**Fig. 1.**
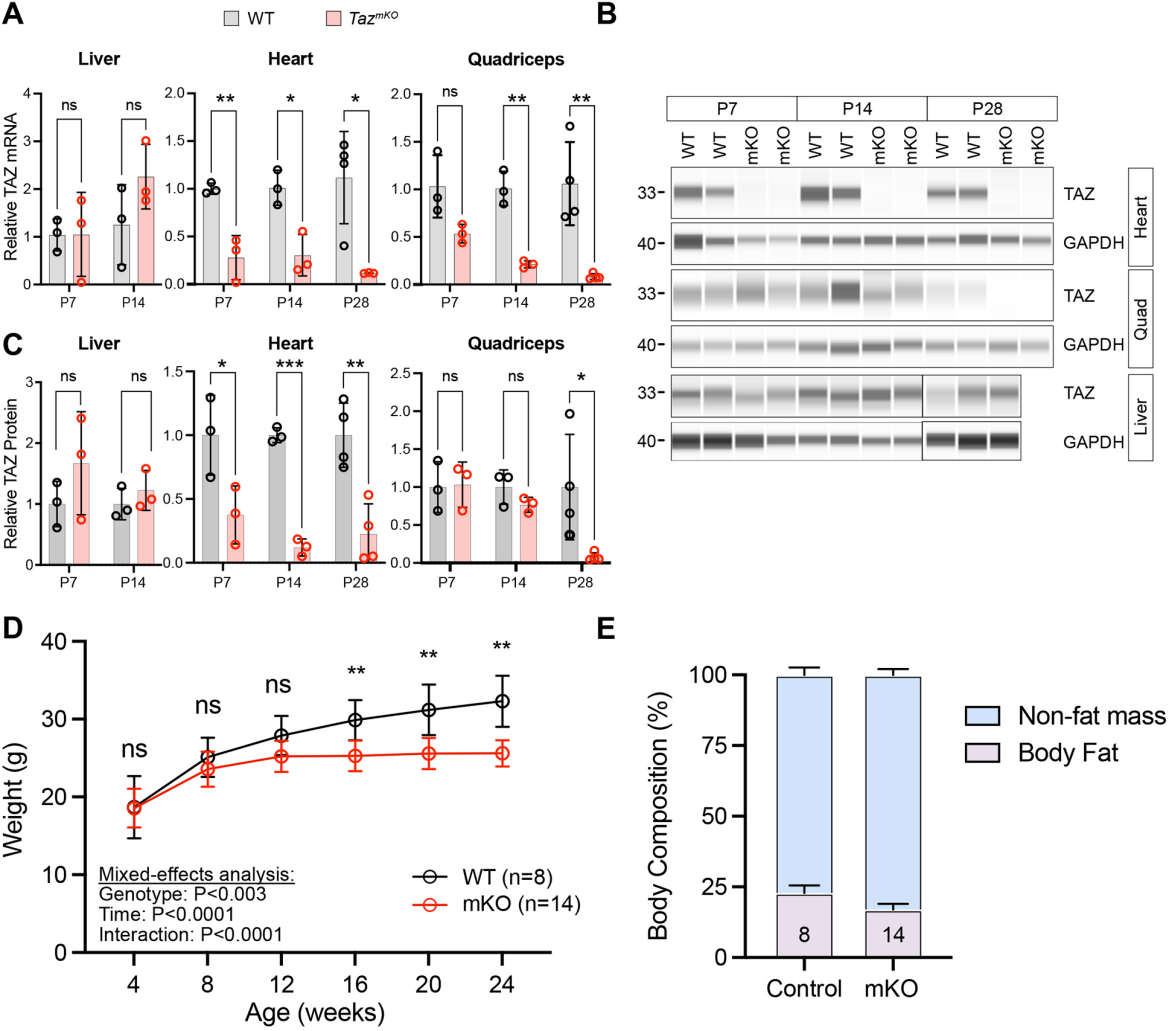
***Taz* knockout and growth of *Taz^mKO^* and control mice.** (A) Reverse transcription quantitative PCR (RT-qPCR) measurement of *Taz* and *Gapdh* mRNA levels. Unpaired two-tailed *t*-test. (B,C) Capillary western blot measurement of Cre, TAZ and GAPDH protein levels at postnatal day (P)7, P14, and P28. Representative blots are shown in B. Quantification is shown in C. Unpaired two-tailed *t*-test. (D) Serial measurement of the weight of *Taz^mKO^* (mKO) and littermate control (WT) mice. Mixed-effects model with Sidak’s multiple comparison test. (D) Assessment of body composition by DEXA scan at 6 months of age. Unpaired two-tailed *t*-test. ns, not significant; *P<0.05, **P<0.01, ***P<0.001. Graphs show mean±s.d. Data points in A and C represent samples from different animals.

We crossed *mCK-Cre* male and *Taz^fl/fl^* female mice to obtain *Taz^fl/Y^; mCK-Cre* (abbreviated as *Taz^mKO^*) and control (*Taz^fl/Y^* without *mCK-Cre*) littermates. We performed reverse transcription quantitative PCR (RT-qPCR) and capillary western blotting to assess the timing and extent of *Taz* knockout at postnatal day (P)7, P14 and P28 (Fig. 1A). In heart, *Taz* mRNA was significantly depleted already at P7. In skeletal muscle, *Taz* mRNA became significantly depleted at P14. In contrast, *Taz* mRNA was not significantly affected in the liver.

We measured depletion of TAZ protein by capillary western blotting (Fig. 1B) and quantified the reduction in TAZ protein (Fig. 1C). TAZ protein was markedly reduced in *Taz^mKO^* heart by P7. In skeletal muscle, TAZ protein ablation was not evident until P28. TAZ protein was unaffected in the liver. Together, these studies confirm the selective inactivation of *Taz* in *Taz^mKO^* cardiac and skeletal muscle.

Unlike germline *Taz-KO* mice on the C57BL/6J background (Wang et al., 2020), *Taz^mKO^* mice were born at the expected Mendelian frequency, and survival to 6 months of age, the oldest age examined, was normal. Also unlike germline *Taz-KO* mice, *Taz^mKO^* mice showed a normal increase in body weight compared to the controls at 4, 8 and 12 weeks of age (Fig. 1D). *Taz^mKO^* mice subsequently maintained a relatively stable weight, whereas littermate controls continued to gain weight so that by 24 weeks of age *Taz^mKO^* mice had 20% lower weight. Tibia length was comparable between genotypes (17.8±0.4 mm control versus 17.9±0.4 mm *Taz^mKO^*, *n*=8 and 14, respectively), indicating that the weight difference is not due to a difference in linear growth. We performed DEXA scanning to measure body mass composition. *Taz^mKO^* mice had a significantly reduced proportion of fat compared to non-fat mass (Fig. 1B). After factoring in this difference in body fat, the calculated non-fat mass of *Taz^mKO^* mice remained lower than that of controls (21.3 g versus 24.9 g). Even though *Taz* depletion was limited to striated muscle, *Taz^mKO^* mice also had reduced fat mass, suggesting a non-cell autonomous effect of ablation of striated muscle *Taz* on the amount of adipose tissue.

### Cardiac phenotype of conditional *Taz^mKO^* mice

**Fig. 2.**
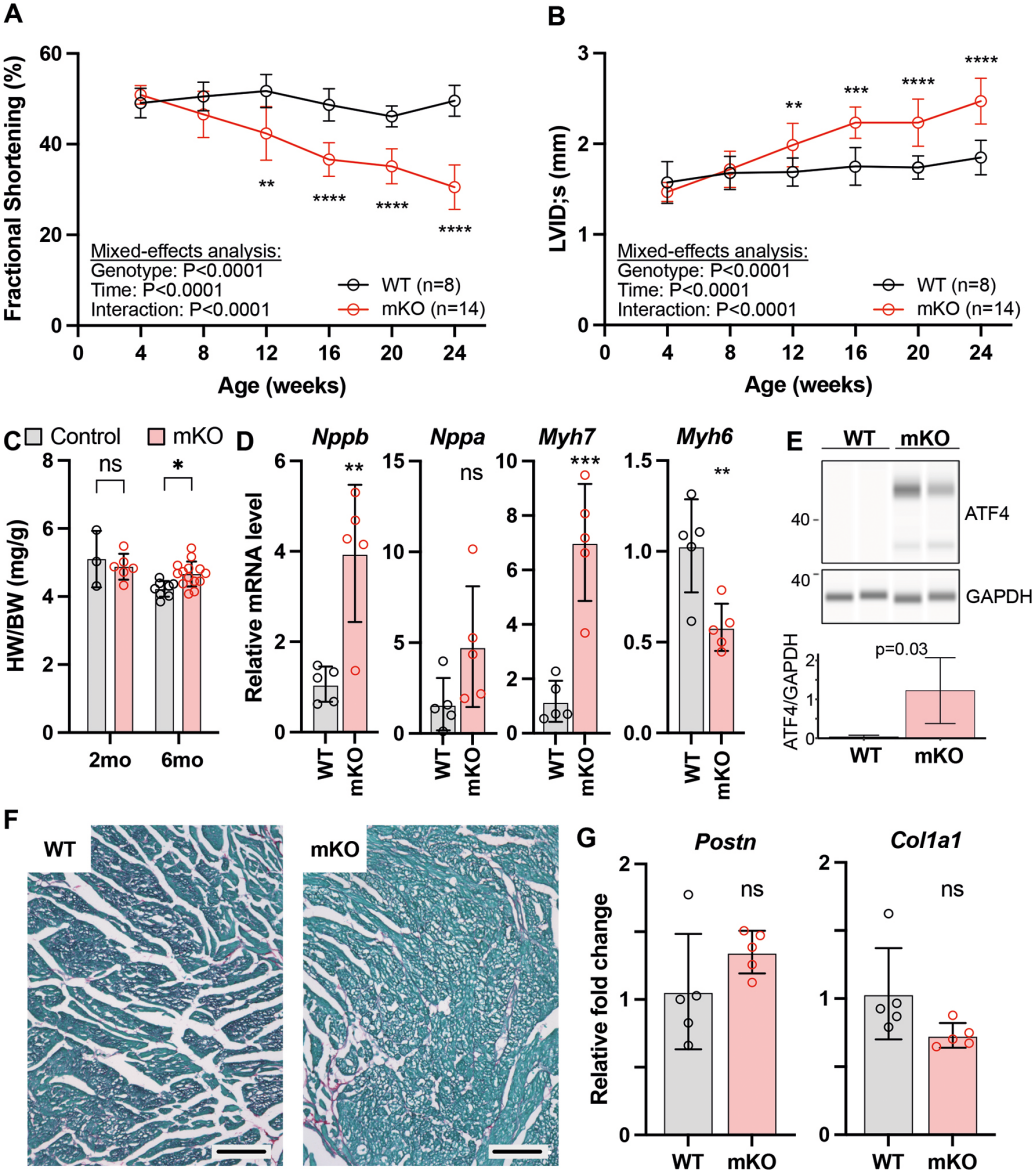
**Systolic heart failure in *Taz^mKO^* mice.** (A,B) Echocardiographic measurements of heart systolic function [fractional shortening (%); A] and left ventricular internal diameter at end systole (LVID;s; B) were made monthly from 1 to 6 months of age. Mixed-effects analysis with repeated measures Sidak's multiple comparison test. (C) Heart weight (HW) normalized to body weight (BW) at 2 and 6 months of age. Unpaired two-tailed *t*-test. (D) Relative mRNA levels of the indicated cardiac stress markers, as measured by RT-qPCR from ventricular tissue at 6 months of age. Data are normalized to *Gapdh*. Unpaired two-tailed *t*-test with Holm-Sidak multiple testing correction. (E) Cardiac ATF4 protein levels. Top: representative capillary western blot of ATF4 and GAPDH. Lower and upper band are consistent with ATF4 and phosphorylated ATF4. Bottom: quantitative comparison of ATF4 normalized to GAPDH. *n*=5. (F) Cardiac sections stained with Picrosirus Red and Fast Green. Scale bars: 100 µm. (G) Relative mRNA levels of cardiac fibrosis markers. Unpaired two-tailed *t*-test. Data are presented as mean±s.d. Each point in C, D and G represents one mouse. ns, not significant; *P<0.05, **P<0.01, ***P<0.001, ****P<0.0001.

Most patients with Barth syndrome have cardiomyopathy, leading to infant mortality and heart transplantation in ∼15% of patients (Chowdhury et al., 2022; Roberts et al., 2012). To assess cardiac function, *Taz^mKO^* and control mice underwent echocardiography monthly from 1 to 6 months of age. Cardiac dysfunction, measured echocardiographically by left ventricular fractional shortening, became statistically significant at 3 months of age (Fig. 2A; Fig. S1). With increasing age, systolic heart function continued to decline, whereas that of controls remained relatively stable. Mutant mice had increased left ventricular internal diameter at end systole starting at the age of 3 months, consistent with contractile dysfunction and a systolic heart failure phenotype (Fig. 2B).

After euthanasia, we measured heart weight and normalized it to body weight (HW/BW ratio). At the age of 2 months, the HW/BW ratio was similar between *Taz^mKO^* mice and littermate controls; however, by 6 months of age, the *Taz^mKO^* HW/BW ratio was significantly higher (Fig. 2C). RNA levels of cardiac stress markers *Nppb* and *Myh7* were significantly elevated in *Taz^mKO^* mice compared to controls (Fig. 2D). *Nppa* was also higher in *Taz^mKO^* mice than in controls but did not reach statistical significance. *Myh6*, the major cardiac myosin isoform in mice, was significantly decreased. Together, these data indicate that *Taz^mKO^* mice develop systolic heart failure.

We and others previously demonstrated that *Taz* deficiency strongly activates the integrated stress response, with signature changes found in both the transcriptome and metabolome (Kutschka et al., 2023; Wang et al., 2023; Zhu et al., 2022). An important driver of this response is post-transcriptional upregulation of ATF4, a transcription factor that mediates many of the gene expression changes observed in the integrated stress response. We observed strong upregulation of ATF4 protein in *Taz^mKO^* myocardium (Fig. 2E), consistent with the prior studies.

We analyzed tissue sections for evidence of cardiac fibrosis. Picrosirus Red staining did not reveal significantly increased fibrosis in *Taz^mKO^* myocardium (Fig. 2F). Consistent with this observation, collagen (*Col1a1*) and periostin (*Postn*) RNA levels did not differ significantly between groups (Fig. 2G).

### Exercise and skeletal muscle phenotype of *Taz^mKO^* mice

**Fig. 3.**
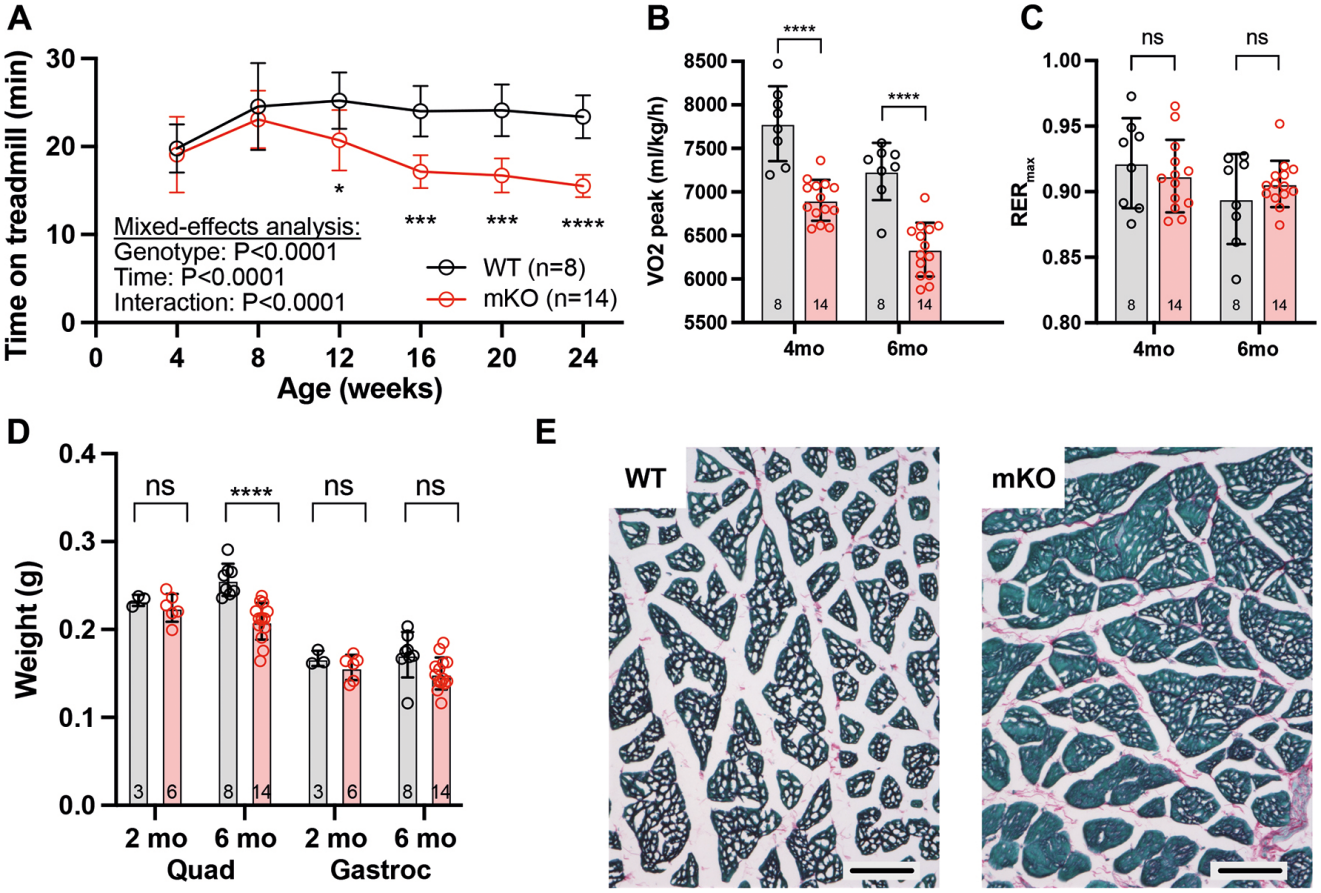
**Treadmill endurance and skeletal muscle characterization of *Taz^mKO^* mice.** (A) Treadmill endurance of *Taz^mKO^* and control mice over time. Mixed effects model with repeated measures. Comparisons between genotypes at each time point was corrected for multiple testing using Sidak's method. (B) Peak oxygen consumption rate (VO_2_) in *Taz^mKO^* and control mice aged 4 and 6 months. Unpaired two-tailed *t*-test. (C) Maximal respiratory exchange ratio (RER_max_) in *Taz^mKO^* and control mice aged 4 and 6 months. Unpaired two-tailed *t*-test. (D) Skeletal muscle weights in mice aged 2 and 6 months. Unpaired two-tailed *t*-test. (E) Representative Picrosirus Red/Fast Green staining of quadriceps and skeletal muscle at 6 months. Scale bars: 100 µm. Graphs show mean±s.d. Each point represents one mouse. ns, not significant; *P<0.05, ***P<0.001, ****P<0.0001.

Skeletal muscle weakness and low endurance is a prominent symptom of patients with Barth syndrome (Bowen et al., 2019). Skeletal muscle function can be measured by treadmill exercise testing (Bashir et al., 2017; Spencer et al., 2011). The skeletal muscle performance of *Taz^mKO^* mice was assessed by measuring treadmill endurance monthly on a metabolic treadmill. By the age of 3 months, *Taz^mKO^* mice had lower treadmill endurance than littermate controls, and endurance progressively declined thereafter (Fig. 3A). Using a metabolic treadmill, we monitored oxygen consumption (VO_2_) and carbon dioxide production (VCO_2_) rates, and the respiratory exchange ratio (RER=VCO_2_/VO_2_). Peak oxygen consumption was significantly decreased in mutant mice at 4 and 6 months of age (Fig. 3B). The RER at exhaustion did not significantly differ between genotypes (Fig. 3C).

We compared quadricep and gastrocnemius muscle weights at 2 and 6 months of age (Fig. 3D). At 2 months, muscle weights were comparable. At 6 months of age, *Taz^mKO^* quadricep muscles had significantly lower mass than that of controls, whereas gastrocnemius weights did not significantly differ.

To assess skeletal muscle fibrosis, we performed Picrosirius Red staining of quadricep muscles at 6 months of age. We observed no increase in fibrosis in *Taz^mKO^* muscles (Fig. 3E).

### Metabolic assessment of *Taz^mKO^* mice under normal cage activity

**Fig. 4.**
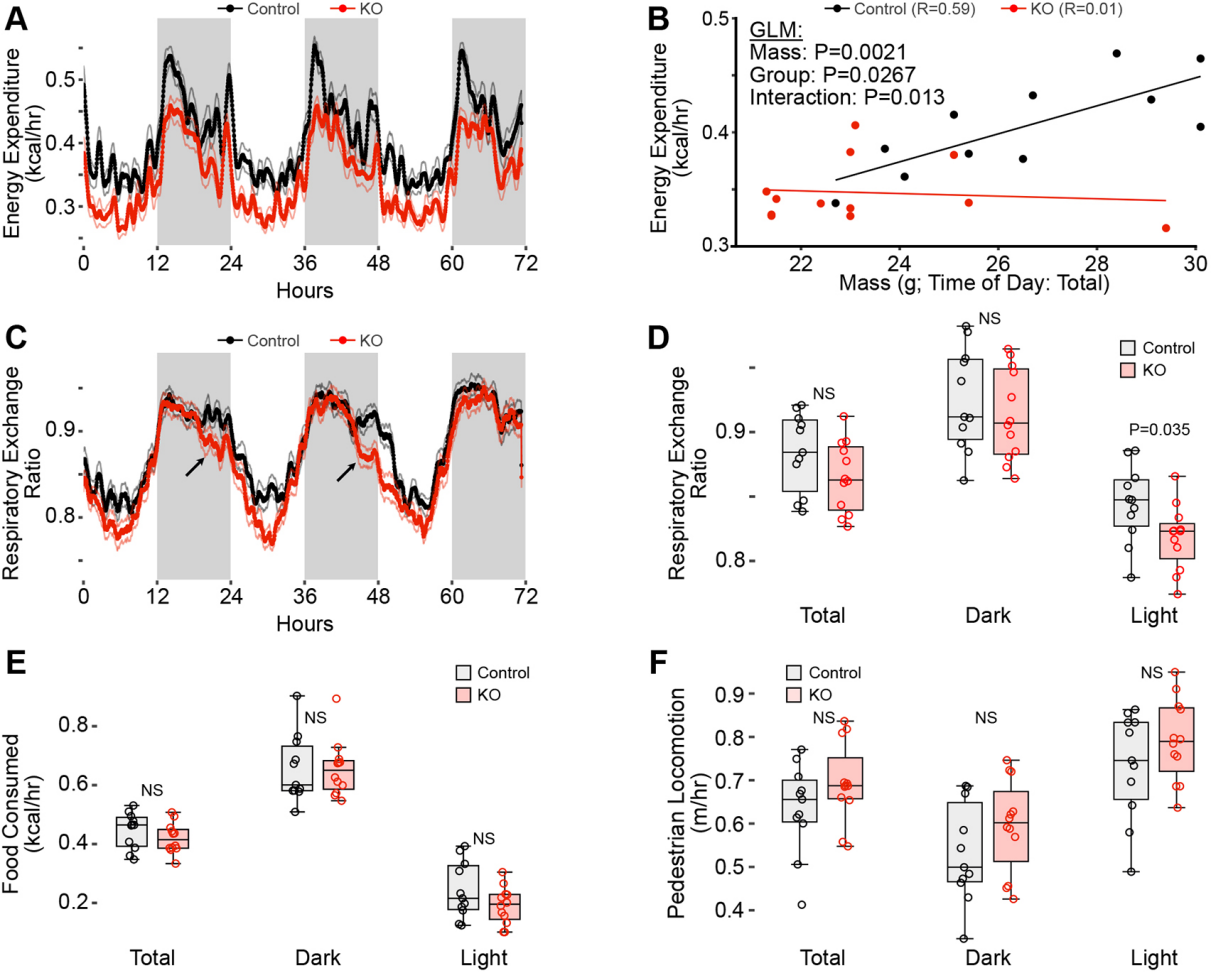
**Metabolic activity of *Taz^mKO^* and control mice with normal cage activity.** Eleven control and 13 *Taz^mKO^* mice aged 4-5 months were individually housed, and VO_2_, VCO_2_, activity and food intake were monitored for 84 h. (A) Total energy over time. (B) Total energy expenditure versus mouse weight. Analysis using a generalized linear model (GLM) showed significant effects of mass and genotype, and a significant interaction between mass and genotype. (C) Respiratory exchange ratio (RER) over time. Arrows point to later-dark photoperiod with lower RER. (D) RER in dark or light photoperiods, or total. One-way ANOVA. (E,F) Food intake (E) and activity (F) in dark or light photoperiods, or total. One-way ANOVA. In A and C, heavy and light lines indicate mean and s.e.m., respectively. Arrows indicate difference in RER near the end of the dark cycle. Each point in B, D, E and F represents one mouse. White and gray shading indicate light and dark photoperiods, respectively. In B, D, E and F, the central line, box and whiskers indicate the median, 25th and 75th percentile, and 1.5× the interquartile range, respectively. NS, not significant.

To further characterize the *Taz^mKO^* mice, we performed indirect calorimetry on 4- to 5-month-old mice and, at the same time, monitored food intact and activity level. Mice were acclimated to the system for 31 h and then monitored for 84 h. Energy expenditure was higher in control mice than in *Taz^mKO^* mice (Fig. 4A). To account for the higher weight of control mice, we analyzed the relationship between energy expenditure and weight. In control mice, energy expenditure was proportional to body weight, as expected; in *Taz^mKO^* mice, energy expenditure did not vary with body weight (Fig. 4B), perhaps owing to low energy expenditure by mutant muscle tissue. Surprisingly, *Taz^mKO^* mice had decreased RER during the light photoperiod, suggestive of increased fatty acid utilization (Fig. 4C,D), which may contribute to their reduced body fat (Fig. 1E). RER did not significantly differ between genotypes during the dark photoperiod, although RER appeared lower in mutants than in controls in the late-dark photoperiod (Fig. 4C, arrows), when mice have reduced activity and food intake compared to in the early-dark photoperiod. Food intake and activity levels did not significantly differ between genotypes (Fig. 4E,F).

### Abnormal cardiolipin composition of *Taz^mKO^* striated muscles

**Fig. 5.**
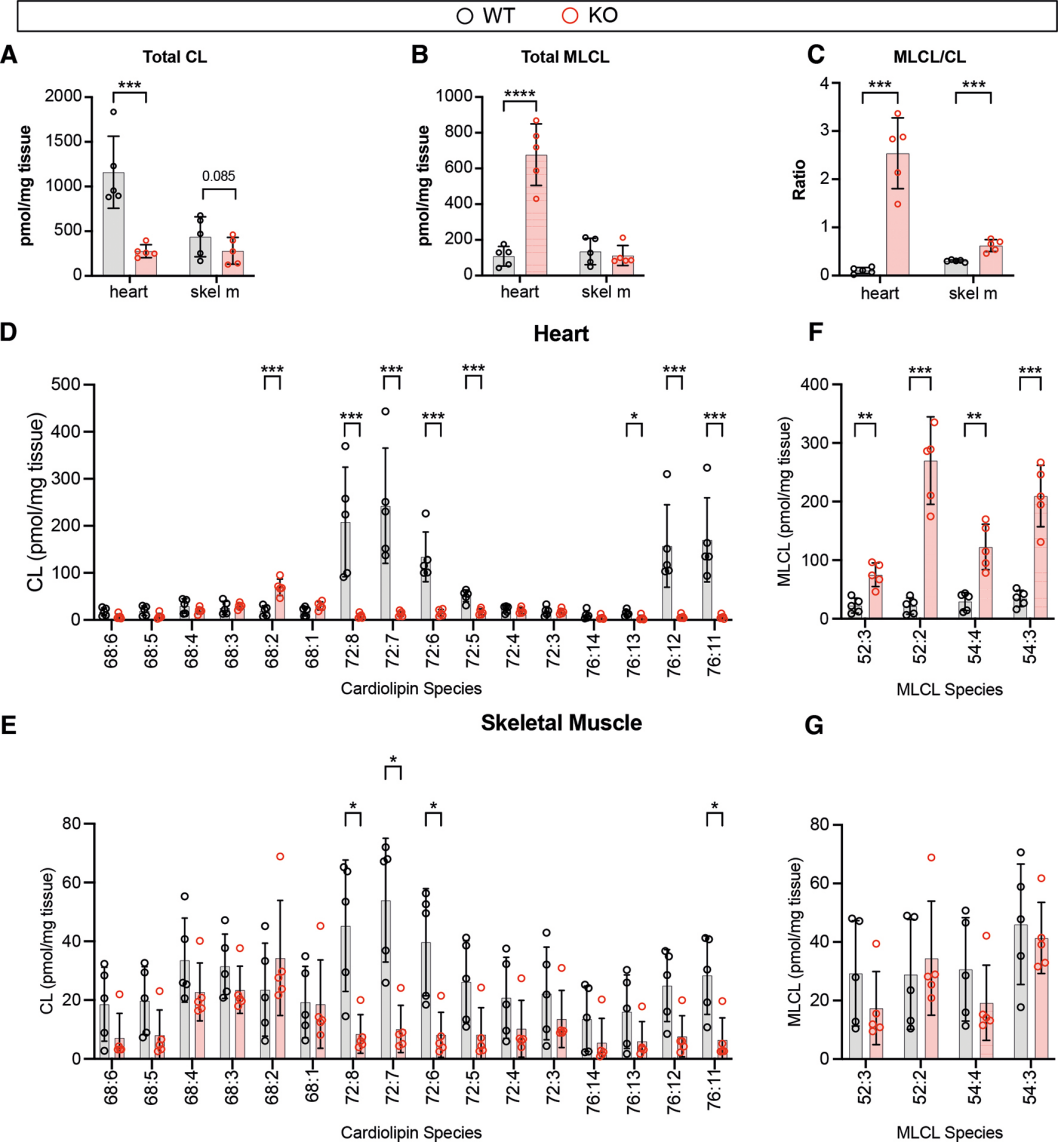
**Cardiolipin composition of *Taz^mKO^* cardiac and skeletal muscle.** Cardiolipin composition was measured using matrix-assisted laser desorption/ionization-time of flight mass spectrometry with an internal standard. (A-C) Total cardiolipin (CL; A), monolysocardiolipin (MLCL; B) and MLCL/CL ratio (C) of cardiac and skeletal muscle (quadriceps). (D-G) Distribution of CL (D,E) and MLCL (F,G) species in heart (D,F) and quadriceps muscle (E,G). Graphs show mean±s.d. Each point represents a separate mouse. Unpaired two-tailed *t*-test with false discovery rate (FDR) multiple-testing correction. FDR with values less than 0.05 are shown. *P<0.05, **P<0.01, ***P<0.001, ****P<0.0001.

TAZ catalyzes the biogenesis of mature cardiolipin (CL) (Schlame and Greenberg, 2017), which, in cardiac muscle, primarily contains four 18:2 acyl chains (tetralinoleoyl CL). TAZ deficiency disrupts normal CL remodeling, resulting in lower total CL, elevated monolysocardiolipin (MLCL) and atypical acyl chain composition (Schlame and Greenberg, 2017). We used mass spectrometry to measure the CL and MLCL composition of 6-month-old *Taz^mKO^* cardiac and quadricep muscles. *Taz^mKO^* cardiac muscles had the low CL, high MLCL and high MLCL/CL ratio that is the hallmark of TAZ deficiency (Fig. 5A-C). *Taz^mKO^* skeletal muscle had a trend towards lower CL, no significant difference in MLCL and a significantly elevated MLCL/CL ratio (Fig. 5A-C). These changes in CL, MLCL and MLCL/CL in skeletal muscle were more mild than has been reported previously in skeletal muscle of mice with widespread *Taz* knockdown (Acehan et al., 2011) or in patients with Barth syndrome (Houtkooper et al., 2009). This difference might be due to differences in species, strain, muscles sampled and potentially incomplete *Taz* inactivation in *Taz^mKO^* mice.

CL and MLCL species differ by their acyl chain composition. We compared the relative abundance of individual CL species in heart (Fig. 5D) and skeletal muscle (Fig. 5E). Control myocardium showed the expected strong enrichment for tetralinoleoyl CL (four 18:2 acyl chains=72:8) as well as 72:7 (Fig. 5D). In *Taz^mKO^* myocardium, these species were depleted. The most abundant CL species in mutant heart was 68:2, which was elevated compared to that in control heart. Control skeletal muscle tissue showed a wider range of CL species, with 72:8 and 72:7 being the most abundant. *Taz^mKO^* skeletal muscle was depleted for these isoforms (Fig. 5E). The most abundant CL species in mutant skeletal muscle were 68:4, 68:3 and 68:2, which were comparable in abundance to in controls.

Each MLCL species was present at low levels in control heart and elevated in *Taz^mKO^* heart (Fig. 5F). In skeletal muscle, the abundance of each MLCL species was comparable between mutant and control (Fig. 5G).

Together, these observations indicate that *Taz^mKO^* skeletal and cardiac muscle have CL abnormalities consistent with *Taz* deficiency.

### Abnormal mitochondrial morphology and distribution in *Taz^mKO^* striated muscles

**Fig. 6.**
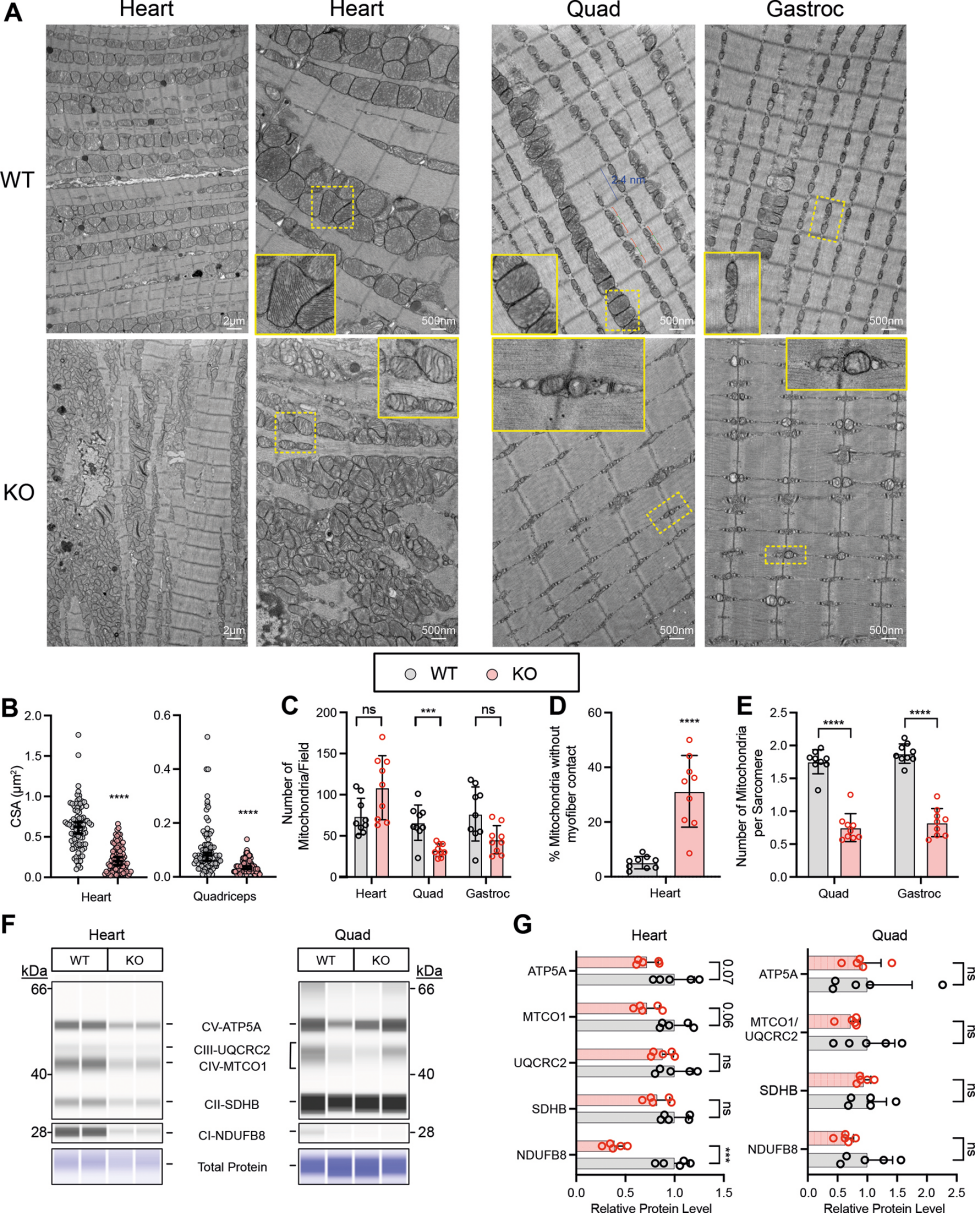
***Taz^mKO^* mitochondrial morphology.** (A) Representative electron micrographs of 6-month-old *Taz^mKO^* and littermate control ventricular myocardium, quadriceps muscle (Quad) and gastrocnemius muscle (Gastroc). Dashed line boxed areas are enlarged in insets. (B) Mitochondrial cross-sectional area (CSA). Each point represents one mitochondrion. (C) Mitochondrial number density. Each point represents one field. (D) Clumping of cardiac mitochondria, scored as the percentage of mitochondria without myofiber contact. Each point represents one field. Mitochondrial clustering was not observed in skeletal muscles and was not quantified. (E) Number of mitochondria per sarcomere in skeletal muscles. Each point represents one field. Quantification in B-E was based on three images per mouse with three mice per group. B indicates median±95% c.i., while bar graphs in C and D show mean±s.d. Unpaired two-tailed *t*-test. (F) Representative capillary western blots of proteins from respiratory complexes I-V. (G) Quantification of F. In Quad, MTCO1 and UQCRC2 were not well resolved and were quantified together. Results were normalized to total protein, measured by intensity of Coomassie Blue staining across the entire capillary. *n*=5. Each point represents one mouse sample. Unpaired two-tailed *t*-test. ns, not significant (*P*>0.1); ***P<0.001, ****P<0.0001. In B-E, electron microscopy images were obtained from three separate mice.

CL is a critical lipid of the mitochondrial inner membrane that has been implicated in its folding into cristae (Ikon and Ryan, 2017; Joubert and Puff, 2021), and TAZ deficiency causes abnormal mitochondrial morphology, size and number (Acehan et al., 2011; Soustek et al., 2011; Wang et al., 2020, 2023). We used electron microscopy to analyze mitochondrial morphology in *Taz^mKO^* heart and skeletal muscle. Consistent with prior studies of TAZ-deficient cardiac muscle (Acehan et al., 2011; Soustek et al., 2011; Wang et al., 2020, 2023), *Taz^mKO^* cardiac mitochondria had abnormal morphology and organization. The *Taz^mKO^* cardiac mitochondria had highly simplified internal cristae (Fig. 6A) and lower cross-sectional area than those of controls (Fig. 6A,B). The number density of cardiac mitochondria was higher in mutants than in controls, although this difference did not reach statistical significance (Fig. 6A,C). Control cardiac mitochondria were organized so that each sarcomere neighbored and contacted approximately two mitochondria, whereas in *Taz^mKO^* mice the mitochondria appeared piled up, and a significantly higher fraction was not in contact with a sarcomere (Fig. 6A,D).

In skeletal muscle, *Taz^mKO^* mitochondria also had sparse cristae and lower cross-sectional area than controls (Fig. 6A,B). Unlike in cardiac muscle, in skeletal muscle, the number of mitochondria was lower than that in controls, with the difference being significant in quadriceps and tending towards significance in gastrocnemius (Fig. 6C). Also different from cardiac muscle, mitochondria did not form disorganized clumps in *Taz^mKO^* skeletal muscle (Fig. 6A). Mitochondria remained localized near Z-lines, but the average number of mitochondria per sarcomere was lower in *Taz^mKO^* skeletal muscles than in control skeletal muscles (Fig. 6E).

To further characterize the effect of TAZ depletion on mitochondria, we measured the level of proteins in respiratory complexes I-V in the heart and quadriceps (Fig. 6F,G). In the heart, the level of complex I protein NDUFB8 was significantly lower in *Taz^mKO^* mice than in control mice, consistent with prior studies of hearts from *Taz* knockdown mice (Le et al., 2020) and patients with Barth syndrome (Chatfield et al., 2022). In the quadriceps, we did not observe a significant difference in the levels of the measured respiratory complex proteins.

## DISCUSSION

We characterized a new Barth syndrome mouse model in which a floxed *Taz* allele is inactivated by *mCK-Cre*, a well-characterized Cre allele selectively active in cardiac and skeletal muscle (Brüning et al., 1998). *mCK-Cre* regulatory elements turn on late in embryogenesis and in the first postnatal week (Brüning et al., 1998). Consistent with this timing, *Taz* transcript levels declined from P7 to P14, and TAZ protein was significantly decreased by P7 in heart and by P28 in skeletal muscle. *Taz^mKO^* mice survived normally and had normal size through 8 weeks of age, unlike germline *Taz-KO* mice, which are significantly smaller from birth (Wang et al., 2020). *Taz^mKO^* mice subsequently had lower body weight than their control counterparts. *Taz^mKO^* mice also had significantly reduced fat mass compared to control littermates, indicating that *Taz* expression in striated muscle has cell non-autonomous effects on the size of the adipose compartment. This might be due to increased fatty acid oxidation during light photoperiods, which we detected by indirect calorimetry. Because *Taz* is required for efficient fatty acid oxidation, increases in fatty acid oxidation likely occur in *Taz*-replete, non-muscle tissue such as adipose tissue.

Likely owing to *Taz* inactivation in the early postnatal period, *Taz^mKO^* mice survived normally to adulthood. *Taz^mKO^* had progressive cardiac dysfunction. The degree of systolic dysfunction was less severe than that of either *Taz-KO* mice or *Taz^fl/Y^; Myh6-Cre* cardiac-specific knockout mice. Furthermore, we did not detect substantial cardiac fibrosis, unlike in *Taz-KO* or *Taz^fl/Y^; Myh6-Cre* models (Wang et al., 2020, 2023). This could be due to later timing or possibly lower extent of cardiomyocyte *Taz* inactivation by mCK-Cre. *Taz^mKO^* mice also had progressive declines in treadmill endurance, with an associated reduction in maximal VO_2_. The phenotype was more mild than that of *Taz-KO* mice in the same C57BL/6J genetic background (Wang et al., 2020, 2023), again possibly owing to later or less complete *Taz* inactivation. Because we have observed that *Taz-KO* mice in the CAST-C57BL/6J F1 background have severe cardiac dysfunction yet normal treadmill endurance (Wang et al., 2023), it is most likely that reduced treadmill endurance in *Taz^mKO^* mice is a skeletal muscle phenotype, rather than a result of impaired cardiac function. We previously noted stark differences in the phenotypes of *Taz-KO* mice in different strain backgrounds, with C57BL/6J mice exhibiting the most severe combination of cardiac and skeletal muscle phenotypes (Wang et al., 2023). This study also used the C57BL/6J strain, and it is likely that the phenotypes observed would be weaker in other strain backgrounds.

*Taz^mKO^* muscles exhibited hallmark biochemical features of *Taz* deficiency, including upregulation of ATF4, a marker of the integrated stress response (Kutschka et al., 2023; Wang et al., 2023; Zhu et al., 2022) and abnormal cardiolipin composition (Schlame and Greenberg, 2017). Associated with these cardiolipin abnormalities, *Taz^mKO^* muscles contained small, disorganized mitochondria with reduced cristae, consistent with mitochondrial abnormalities previously noted in *Taz-KO* cardiomyocytes (Acehan et al., 2011; Soustek et al., 2011; Wang et al., 2020, 2023). In addition to these similarities between *Taz^mKO^* heart and skeletal muscle mitochondria, we also observed differences. In the heart, the number of mitochondria per field tended to be higher than in controls (although this did not reach statistical significance), and mitochondria bunched together and lost their characteristic alignment next to sarcomeres. In the quadriceps, the number of mitochondria per field was significantly reduced, but mitochondria retained their characteristic alignment next to sarcomeres. Our analysis of *Taz-KO* mice in different genetic backgrounds suggested a correlation between cardiac dysfunction and impaired mitophagy (Wang et al., 2023), and we suspect that the abnormal organization and number of cardiac mitochondria reflects altered mitochondrial dynamics and quality control, which may be restricted to cardiomyocytes.

It is important to recognize that *Taz^mKO^* mice do not mimic all aspects of Barth syndrome. For instance, patients with Barth syndrome have considerable heterogeneity in clinical manifestations, with a subset having severe neonatal cardiomyopathy that requires transplantation (Taylor et al., 2022). This aspect of Barth syndrome is not well modeled in *Taz^mKO^* mice or even in *Taz-KO* [C57BL/6J] mice (Wang et al., 2020), which do have neonatal cardiac dysfunction. Patients with Barth syndrome (Spencer et al., 2011) exhibit markedly elevated RER during exercise, and mice with widespread *Taz* knockdown also had elevated RER at peak exercise (Powers et al., 2013). In contrast, RER did not significantly differ between control and *Taz^mKO^* mice at peak exercise. Under normal cage activity, RER was even significantly lower in *Taz^mKO^* mice during light photoperiods, unlike mice with widespread *Taz* knockdown, which had unchanged RER during light photoperiods and elevated RER during dark photoperiods (Goncalves et al., 2021). This difference suggests that *Taz*-deficient striated muscles stimulate increased fatty acid oxidation in other *Taz*-replete tissues in the *Taz^mKO^* model. Perhaps related to the divergent RER responses between patients with Barth syndrome and *Taz^mKO^* mice, the exercise intolerance of *Taz^mKO^* mice is comparatively mild, with no difference in activity under normal cage activity and a modest (∼33%), but significant, reduction in endurance with forced treadmill running. Finally, we noted more mild abnormalities of cardiolipin composition in *Taz^mKO^* skeletal muscle than in prior studies of Taz knockdown mice or patients with Barth syndrome, which might be due to differences in species, strain, muscles sampled and potential for incomplete *Taz* inactivation in the *Taz^mKO^* mice. This difference may contribute to a more mild skeletal muscle phenotype than that observed in *Taz^KO^* [C57BL/6J] mice that survive to adulthood (Wang et al., 2020, 2023) and should be kept in mind when using the *Taz^mKO^* model.

Despite these limitations, the *Taz^mKO^* mouse models aspects of *Taz* deficiency in striated muscles and is a suitable pre-clinical model to test the efficacy of novel therapies designed to the treat the heart and skeletal muscle manifestations of *Taz* deficiency in Barth syndrome.

## MATERIALS AND METHODS

### Mice

Animal experiments were performed under protocols approved by the Boston Children's Hospital Institutional Animal Care and Use Committee. The mice were housed in groups of two to five per cage in an animal facility with exposure to a standard diet. *mCK-Cre* transgenic (Brüning et al., 1998) male mice were bred with *Taz^fl/fl^* females, in which exons 5-10 are flanked by loxP sequences (Ren et al., 2019; Wang et al., 2020). Litters were genotyped for *mCK-Cre* and *Taz^fl^*, and males were used for the experiments described in this paper. Genotyping was performed by PCR analysis of toe clip genomic DNA using the primers listed in Table S1.

### Treadmill exercise test

We measured the exercise capacity of mice using an incremental exercise test on a metabolic treadmill, which provides gas exchange measurements, including oxygen consumption (VO_2_) and carbon dioxide production (VCO_2_) rates normalized to mouse body weight (Columbus Instruments). Mice were placed in an enclosed chamber containing a motorized treadmill with adjustable speed and incline. The treadmill was equipped with an electrified metal grid at the end of the moving belt to provide motivation for mice to run rather than rest on the grid. Electric shock intensity was set to 0.08 mA. Animals were all familiarized with the treadmill prior to the initiation of the protocol. In the test, the mice were recorded at rest for 5 min, then started to run at a 5% incline at 5 m/min, increasing by 5 m/min every 5 min until exhaustion. The shock grid was turned off or the treadmill was stopped for exhaustion if the mice stayed on the shock grid for over 5 s or if they stayed on the shock grid for 2 s or more five times. Gas samples were collected and analyzed by the Oxymax system throughout the experiment. The time mice spent running on the treadmill was recorded manually.

### Tissue collection

Mice were sacrificed at 1 and 6 months. Samples of heart muscle and skeletal muscles (quadriceps and gastrocnemius) were collected. The body weight, absolute weights of collected tissues and tibial lengths were recorded. Samples were collected, fixed and stored as appropriate.

### DEXA scanning

DEXA scanning (Piximus) was performed under isoflurane anesthesia to collect body composition measurements, including fat and non-fat tissue.

### Echocardiography

Echocardiography was performed monthly in unsedated mice using a Vevo 2100 (VisualSonics). After removal of chest hair, mice were held in a standard hand grip. M-mode short-axis images were acquired for heart function measurements. Measurements included heart rate, fractional shortening, left ventricular internal diameter at end diastole, left ventricular internal diameter at end systole, left ventricular posterior wall at end diastole and left ventricular posterior wall at end systole. Echocardiography was performed unaware of genotype.

### Indirect calorimetry

Mice were housed at 23°C in a Promethion indirect calorimetry system (Sable Systems International) within a temperature-controlled cabinet. Data collected include VO_2_, VCO_2_, physical activity beam breaks, food intake and body mass. For the duration of the experiment, the mice had *ad libitum* access to Labdiet 5008 (3.56 kcal/g) and were maintained on a 12 h/12 h photoperiod with lights on from 06:00 to 18:00. Data were analyzed with CalR2 (Mina et al., 2018).

### Histology

Cardiac and skeletal muscle samples were collected and fixed overnight in 4% paraformaldehyde (PFA) at 4°C with rotation. Tissue samples were paraffin embedded, sectioned and stained with Picrosirius Red/Fast Green. Images were captured at 20× magnification using a wide-field Keyence microscope.

### Electron microscopy

Samples were fixed in EM fixative (1.25% paraformaldehyde, 2.5% glutaraldehyde and 0.03% picric acid in 0.1 M sodium cacodylate buffer, pH 7.4) overnight at 4°C. After fixation, tissues washed in 0.1 M sodium cacodylate buffer and postfixed with 1% OsO_4_/1.5% KFeCN_6_ for an hour. Samples were then washed in water twice, once in 50 mM maleate buffer pH 5.15 and incubated in 1% uranyl acetate in maleate buffer for 1 h. Samples were then washed in maleate buffer, then water twice, and dehydrated for 10 min each in 50%, 70%, 90%, 100% and 100% ethanol. The samples were then put in propyleneoxide for 1 h and infiltrated overnight in a 1:1 mixture of propyleneoxide and TAAB Epon. The following day, the samples were embedded in TAAB Epon and polymerized at 60°C for 48 h. Then, 80 nm sections were cut on a Reichert Ultracut S microtome and imaged with a JEOL 1200EX transmission electron microscope at 80 kV. Images were recorded with an AMT 2k CCD camera and analyzed using ImageJ.

### Capillary western blotting

Cardiac and skeletal muscles were collected and snap frozen. Tissues were homogenized in RIPA buffer at 25 Hz for 2 min using a Qiagen TissueLyser II, incubated for 10 min, then centrifuged at 10,000 ***g*** for 10 min at 4°C, and the supernatant was collected. Protein concentrations were obtained using a Pierce BCA Protein Assay Kit (Thermo Fisher Scientific). Protein quantification was performed using a capillary electrophoresis device (WES, ProteinSimple). Primary antibodies used were as follows: anti-tafazzin mouse monoclonal antibody (mAb), Santa Cruz Biotechnology, sc-365810 (1:40 dilution); anti-Cre-recombinase rabbit mAb, Cell Signaling Technology, 15036S (1:100 dilution); anti-ATF4 rabbit mAb, Cell Signaling Technology, 11815S (1:100 dilution); anti-GAPDH rabbit mAb, Life Technologies, PA116777 (1:100 dilution); and OxPhos Rodent WB Antibody Cocktail (Thermo Fisher Scientific, 45-8099).

### mRNA transcript levels

Tissue samples were collected and snap frozen. Total RNA was purified using TRIzol (Thermo Fisher Scientific). Reverse transcription was performed using Superscript III reverse transcriptase (Life Technologies). RT-qPCR was performed in duplicate for each sample using SYBR Green Master Mix (Thermo Fisher Scientific) and a Bio-Rad CFX96 or CFX384 Touch instrument. Gene expression values were normalized to *Gapdh*. The primer sequences used in this study are listed in Table S1.

### Cardiolipin analysis

Tissue was spiked with cardiolipin mix I (Avanti, LM6003; 30 µl internal standard per 10 mg tissue), and lipids were extracted with chloroform/methanol. Extracted lipids were analyzed by matrix-assisted laser desorption/ionization-time of flight mass spectrometry. Cardiolipin and monolysocardiolipin were expressed as pmol per mg tissue.

### Statistical analysis

Statistical analyses were performed using GraphPad Prism 10 software. All values were expressed as the mean±s.d. The statistical test for each comparison and the number of samples are indicated in the figure legends.

## Supplementary Material

10.1242/dmm.052077_sup1 Supplementary information

## References

[DMM052077C1] Acehan, D., Vaz, F., Houtkooper, R. H., James, J., Moore, V., Tokunaga, C., Kulik, W., Wansapura, J., Toth, M. J., Strauss, A. et al. (2011). Cardiac and skeletal muscle defects in a mouse model of human Barth syndrome. *J. Biol. Chem.* 286, 899-908. 10.1074/jbc.M110.171439 21068380 PMC3020775

[DMM052077C2] Bashir, A., Bohnert, K. L., Reeds, D. N., Peterson, L. R., Bittel, A. J., de Las Fuentes, L., Pacak, C. A., Byrne, B. J. and Cade, W. T. (2017). Impaired cardiac and skeletal muscle bioenergetics in children, adolescents, and young adults with Barth syndrome. *Physiol. Rep.* 5, e13130. 10.14814/phy2.13130 28196853 PMC5309577

[DMM052077C3] Bione, S., D'Adamo, P., Maestrini, E., Gedeon, A. K., Bolhuis, P. A. and Toniolo, D. (1996). A novel X-linked gene, G4.5. is responsible for Barth syndrome. *Nat. Genet.* 12, 385-389. 10.1038/ng0496-385 8630491

[DMM052077C4] Brüning, J. C., Michael, M. D., Winnay, J. N., Hayashi, T., Hörsch, D., Accili, D., Goodyear, L. J. and Kahn, C. R. (1998). A muscle-specific insulin receptor knockout exhibits features of the metabolic syndrome of NIDDM without altering glucose tolerance. *Mol. Cell* 2, 559-569. 10.1016/S1097-2765(00)80155-0 9844629

[DMM052077C5] Chatfield, K. C., Sparagna, G. C., Specht, K. S., Whitcomb, L. A., Omar, A. K., Miyamoto, S. D., Wolfe, L. M. and Chicco, A. J. (2022). Long-chain fatty acid oxidation and respiratory complex I deficiencies distinguish Barth Syndrome from idiopathic pediatric cardiomyopathy. *J. Inherit. Metab. Dis.* 45, 111-124. 10.1002/jimd.12459 34821394

[DMM052077C6] Chowdhury, S., Jackson, L., Byrne, B. J., Bryant, R. M., Cade, W. T., Churchill, T. L., Buchanan, J. and Taylor, C. (2022). Longitudinal observational study of cardiac outcome risk factor prediction in children, adolescents, and adults with barth syndrome. *Pediatr. Cardiol.* 43, 1251-1263. 10.1007/s00246-022-02846-8 35238957 PMC9462389

[DMM052077C7] Clarke, S. L. N., Bowron, A., Gonzalez, I. L., Groves, S. J., Newbury-Ecob, R., Clayton, N., Martin, R. P., Tsai-Goodman, B., Garratt, V., Ashworth, M. et al. (2013). Barth syndrome. *Orphanet J. Rare Dis.* 8, 23. 10.1186/1750-1172-8-23 23398819 PMC3583704

[DMM052077C8] Dudek, J., Cheng, I.-F., Chowdhury, A., Wozny, K., Balleininger, M., Reinhold, R., Grunau, S., Callegari, S., Toischer, K., Wanders, R. J. et al. (2016). Cardiac-specific succinate dehydrogenase deficiency in Barth syndrome. *EMBO Mol. Med.* 8, 139-154. 10.15252/emmm.201505644 26697888 PMC4734842

[DMM052077C9] Goncalves, R. L. S., Schlame, M., Bartelt, A., Brand, M. D. and Hotamışlıgil, G. S. (2021). Cardiolipin deficiency in Barth syndrome is not associated with increased superoxide/H2 O2 production in heart and skeletal muscle mitochondria. *FEBS Lett.* 595, 415-432. 10.1002/1873-3468.13973 33112430 PMC7894513

[DMM052077C10] Houtkooper, R. H., Rodenburg, R. J., Thiels, C., van Lenthe, H., Stet, F., Poll-The, B. T., Stone, J. E., Steward, C. G., Wanders, R. J., Smeitink, J. et al. (2009). Cardiolipin and monolysocardiolipin analysis in fibroblasts, lymphocytes, and tissues using high-performance liquid chromatography-mass spectrometry as a diagnostic test for Barth syndrome. *Anal. Biochem.* 387, 230-237. 10.1016/j.ab.2009.01.032 19454236

[DMM052077C11] Ikon, N. and Ryan, R. O. (2017). Cardiolipin and mitochondrial cristae organization. *Biochim. Biophys. Acta Biomembr.* 1859, 1156-1163. 10.1016/j.bbamem.2017.03.013 28336315 PMC5426559

[DMM052077C12] Joubert, F. and Puff, N. (2021). Mitochondrial cristae architecture and functions: lessons from minimal model systems. *Membranes (Basel)* 11, 465. 10.3390/membranes11070465 34201754 PMC8306996

[DMM052077C13] Kagan, V. E., Tyurin, V. A., Jiang, J., Tyurina, Y. Y., Ritov, V. B., Amoscato, A. A., Osipov, A. N., Belikova, N. A., Kapralov, A. A., Kini, V. et al. (2005). Cytochrome c acts as a cardiolipin oxygenase required for release of proapoptotic factors. *Nat. Chem. Biol.* 1, 223-232. 10.1038/nchembio727 16408039

[DMM052077C14] Kutschka, I., Bertero, E., Wasmus, C., Xiao, K., Yang, L., Chen, X., Oshima, Y., Fischer, M., Erk, M., Arslan, B. et al. (2023). Activation of the integrated stress response rewires cardiac metabolism in Barth syndrome. *Basic Res. Cardiol.* 118, 47. 10.1007/s00395-023-01017-x 37930434 PMC10628049

[DMM052077C15] Le, C. H., Benage, L. G., Specht, K. S., Li Puma, L. C., Mulligan, C. M., Heuberger, A. L., Prenni, J. E., Claypool, S. M., Chatfield, K. C., Sparagna, G. C. et al. (2020). Tafazzin deficiency impairs CoA-dependent oxidative metabolism in cardiac mitochondria. *J. Biol. Chem.* 295, 12485-12497. 10.1074/jbc.RA119.011229 32665401 PMC7458807

[DMM052077C16] Mina, A. I., LeClair, R. A., LeClair, K. B., Cohen, D. E., Lantier, L. and Banks, A. S. (2018). CalR: a web-based analysis tool for indirect calorimetry experiments. *Cell Metab.* 28, 656-666.e1. 10.1016/j.cmet.2018.06.019 30017358 PMC6170709

[DMM052077C17] Pfeiffer, K., Gohil, V., Stuart, R. A., Hunte, C., Brandt, U., Greenberg, M. L. and Schägger, H. (2003). Cardiolipin stabilizes respiratory chain supercomplexes. *J. Biol. Chem.* 278, 52873-52880. 10.1074/jbc.M308366200 14561769

[DMM052077C18] Powers, C., Huang, Y., Strauss, A. and Khuchua, Z. (2013). Diminished exercise capacity and mitochondrial bc1 complex deficiency in tafazzin-knockdown mice. *Front. Physiol.* 4, 74. 10.3389/fphys.2013.00074 23616771 PMC3627988

[DMM052077C19] Pu, W. T. (2022). Experimental models of Barth syndrome. *J. Inherit. Metab. Dis.* 45, 72-81. 10.1002/jimd.12423 34370877 PMC8814986

[DMM052077C20] Ren, M., Xu, Y., Erdjument-Bromage, H., Donelian, A., Phoon, C. K. L., Terada, N., Strathdee, D., Neubert, T. A. and Schlame, M. (2019). Extramitochondrial cardiolipin suggests a novel function of mitochondria in spermatogenesis. *J. Cell Biol.* 218, 1491-1502. 10.1083/jcb.201808131 30914420 PMC6504895

[DMM052077C21] Roberts, A. E., Nixon, C., Steward, C. G., Gauvreau, K., Maisenbacher, M., Fletcher, M., Geva, J., Byrne, B. J. and Spencer, C. T. (2012). The Barth Syndrome Registry: distinguishing disease characteristics and growth data from a longitudinal study. *Am. J. Med. Genet. A* 158A, 2726-2732. 10.1002/ajmg.a.35609 23045169

[DMM052077C22] Schlame, M. and Greenberg, M. L. (2017). Biosynthesis, remodeling and turnover of mitochondrial cardiolipin. *Biochim. Biophys. Acta Mol. Cell Biol. Lipids* 1862, 3-7. 10.1016/j.bbalip.2016.08.010 27556952 PMC5125896

[DMM052077C23] Soustek, M. S., Falk, D. J., Mah, C. S., Toth, M. J., Schlame, M., Lewin, A. S. and Byrne, B. J. (2011). Characterization of a transgenic short hairpin RNA-induced murine model of Tafazzin deficiency. *Hum. Gene Ther.* 22, 865-871. 10.1089/hum.2010.199 21091282 PMC3166794

[DMM052077C24] Spencer, C. T., Byrne, B. J., Bryant, R. M., Margossian, R., Maisenbacher, M., Breitenger, P., Benni, P. B., Redfearn, S., Marcus, E. and Cade, W. T. (2011). Impaired cardiac reserve and severely diminished skeletal muscle O_2_ utilization mediate exercise intolerance in Barth syndrome. *Am. J. Physiol. Heart Circ. Physiol.* 301, H2122-H2129. 10.1152/ajpheart.00479.2010 21873497

[DMM052077C25] Suzuki-Hatano, S., Saha, M., Rizzo, S. A., Witko, R. L., Gosiker, B. J., Ramanathan, M., Soustek, M. S., Jones, M. D., Kang, P. B., Byrne, B. J. et al. (2019). AAV-mediated TAZ gene replacement restores mitochondrial and cardioskeletal function in barth syndrome. *Hum. Gene Ther.* 30, 139-154. 10.1089/hum.2018.020 30070157 PMC6383582

[DMM052077C26] Taylor, C., Rao, E. S., Pierre, G., Chronopoulou, E., Hornby, B., Heyman, A. and Vernon, H. J. (2022). Clinical presentation and natural history of Barth Syndrome: an overview. *J. Inherit. Metab. Dis.* 45, 7-16. 10.1002/jimd.12422 34355402

[DMM052077C27] Thompson, R., Jefferies, J., Wang, S., Pu, W. T., Takemoto, C., Hornby, B., Heyman, A., Chin, M. T. and Vernon, H. J. (2022). Current and future treatment approaches for Barth syndrome. *J. Inherit. Metab. Dis.* 45, 17-28. 10.1002/jimd.12453 34713454

[DMM052077C28] Wang, S., Li, Y., Xu, Y., Ma, Q., Lin, Z., Schlame, M., Bezzerides, V. J., Strathdee, D. and Pu, W. T. (2020). AAV gene therapy prevents and reverses heart failure in a murine knockout model of barth syndrome. *Circ. Res.* 126, 1024-1039. 10.1161/CIRCRESAHA.119.315956 32146862 PMC7233109

[DMM052077C29] Wang, S., Yazawa, E., Keating, E. M., Mazumdar, N., Hauschild, A., Ma, Q., Wu, H., Xu, Y., Shi, X., Strathdee, D. et al. (2023). Genetic modifiers modulate phenotypic expression of tafazzin deficiency in a mouse model of Barth syndrome. *Hum. Mol. Genet.* 32, 2055-2067. 10.1093/hmg/ddad041 36917259 PMC10244222

[DMM052077C30] Zhang, J., Liu, X., Nie, J. and Shi, Y. (2022). Restoration of mitophagy ameliorates cardiomyopathy in Barth syndrome. *Autophagy* 18, 2134-2149. 10.1080/15548627.2021.2020979 34985382 PMC9466615

[DMM052077C31] Zhu, S., Nguyen, A., Pang, J., Zhao, J., Chen, Z., Liang, Z., Gu, Y., Huynh, H., Bao, Y., Lee, S. et al. (2022). Mitochondrial stress induces an HRI-eIF2α pathway protective for cardiomyopathy. *Circulation* 146, 1028-1031. 10.1161/CIRCULATIONAHA.122.059594 36154620 PMC9523491

