# Supplementary material for: A murine model of Barth syndrome recapitulates human cardiac and skeletal muscle phenotypes
Source: Dis Model Mech. 2025 May 19;18(5):dmm052077. doi: 10.1242/dmm.052077 (PMC12128220; doi:10.1242/dmm.052077)
Supplement: Supplementary information [file dmm-18-052077-s1.pdf]

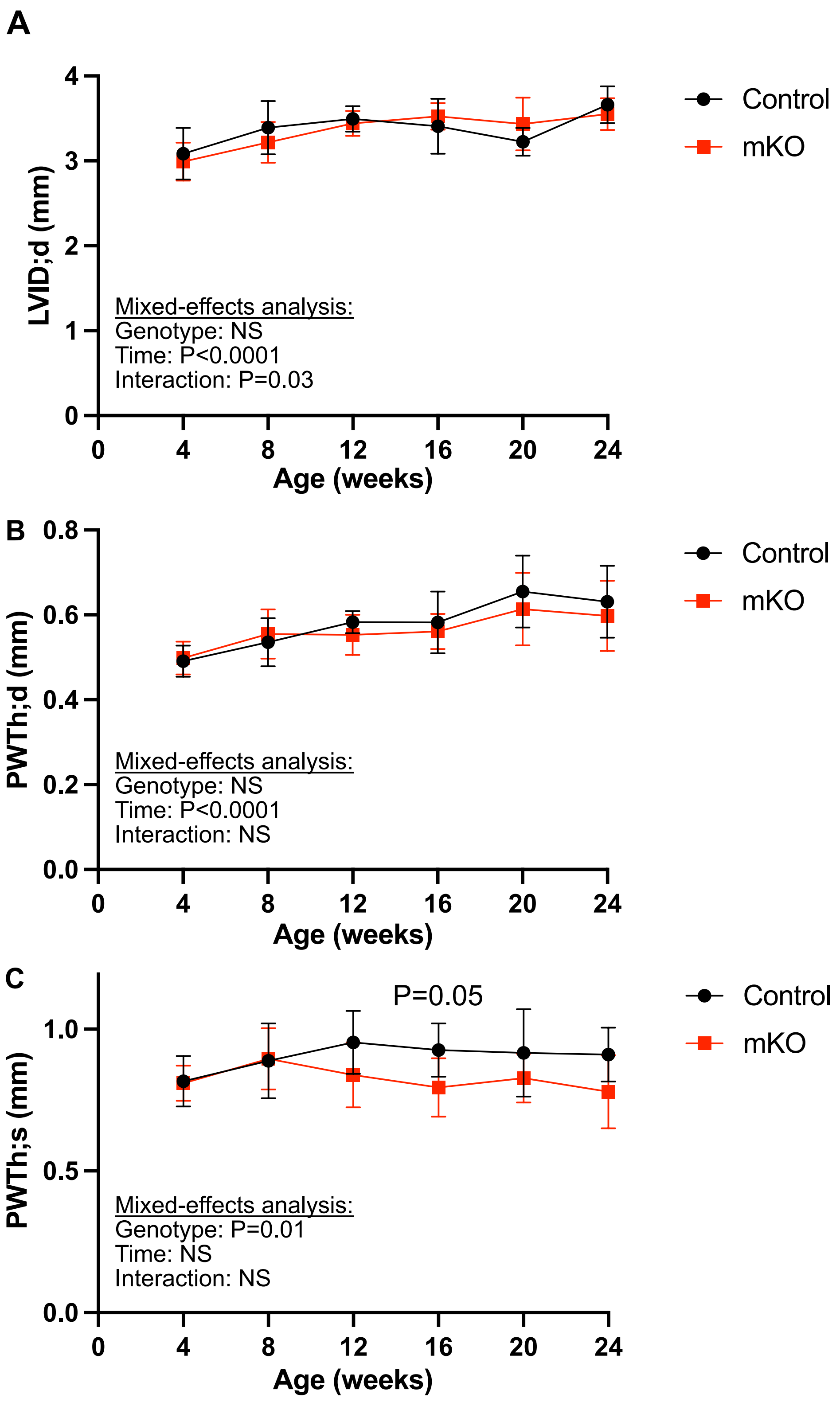

Fig. S1. Cardiac dimensions of control and *Taz*^{mKO} mice. A. LV internal diameter at end diastole. **B.** Posterior wall thickness at end diastole. **C.** Posterior wall thickness at and systole. Repeated measures mixed-effect analysis was performed. Effect of genotype was compared at each time point with Sidek's multiple test correction. Data is represented by mean ± SD. n=9 control and 14 mKO.

Disease Models & Mechanisms • Supplementary information

Table S1. Oligonucleotides used in this study

	RTqPCR		
	Forward	Reverse	
Nppa	GGCCATATTGGAGCAAATCCTGTG	CATGACCTCATCTTCTACCGGCAT	
Nppb	GAGGTCACTCCTATCCTCTGG	GCCATTTCCTCCGACTTTTCTC	
Gapdh	GGAGCGAGATCCCTCCA	GGCTGTTGTCATACTTCTCATGG	
COL1A1	taggccattgtgtatgcagc	acatgttcagctttgtggacc	
Myh7	GCGACTCAAAAAGAAGGACTTTG	GGCTTGCTCATCCTCAATCC	
Myh6	AACCAGAGTTTGAGTGACAGAATG	ACTCCGTGCGGATGTCAA	
Postn	aagctgcggcaagacaag	tcaaatctgcagcttcaagg	
Taz	ATTGGACGGCTGATTGCTGAGTGT	AGTCTGTGAGGGCTTTCCGCATCT	
	Genotyping		
	Primer1	Primer2	Primer3
Taz flox	CTTGCCCACTGCTCACAAAC	CAGGCACATGGTCCTGTTTC	CCAAGTTGCTAGCCCACAAG
mCKCre (Cre)	GTGAAACAGCATTGCTGTCACTT	TAAGTCTGAACCCGGTCTGC	
mCKCre (internal ctrl)	CAAATGTTGCTTGTCTGGTG	GTCAGTCGAGTGCACAGTTT	

Disease Models & Mechanisms • Supplementary information
